# Author Correction: A prospective observational study of post-COVID-19 chronic fatigue syndrome following the first pandemic wave in Germany and biomarkers associated with symptom severity

**DOI:** 10.1038/s41467-022-33784-x

**Published:** 2022-10-12

**Authors:** Claudia Kedor, Helma Freitag, Lil Meyer-Arndt, Kirsten Wittke, Leif G. Hanitsch, Thomas Zoller, Fridolin Steinbeis, Milan Haffke, Gordon Rudolf, Bettina Heidecker, Thomas Bobbert, Joachim Spranger, Hans-Dieter Volk, Carsten Skurk, Frank Konietschke, Friedemann Paul, Uta Behrends, Judith Bellmann-Strobl, Carmen Scheibenbogen

**Affiliations:** 1 Charité - Universitätsmedizin Berlin, corporate member of Freie Universität Berlin and Humboldt Universität zu Berlin, Institute of Medical Immunology, Berlin, Germany; 2 Experimental and Clinical Research Center, a cooperation between the Max Delbrück Center for Molecular Medicine in the Helmholtz Association and Charité Universitätsmedizin Berlin, Berlin, Germany; 3 Experimental and Clinical Research Center, Charité – Universitätsmedizin Berlin, corporate member of Freie Universität Berlin and Humboldt-Universität zu Berlin, Berlin, Germany; 4 Max Delbrück Center for Molecular Medicine in the Helmholtz Association (MDC), Berlin, Germany; 5 Department of Infectious Diseases and Respiratory Medicine, Charité – Universitätsmedizin Berlin, Corporate Member of Freie Universität Berlin and Humboldt-Universität zu Berlin, Berlin, Germany; 6 Department of Cardiology, Charité - Universitätsmedizin Berlin, corporate member of Freie Universität Berlin and Humboldt Universität zu Berlin, Berlin, Germany; 7 Department of Endcrinology and Metabolism, Charité - Universitätsmedizin Berlin, corporate member of Freie Universität Berlin and Humboldt Universität zu Berlin, Berlin, Germany; 8 Center for Regenerative Therapies (BCRT), Berlin Institute of Health, Berlin, Germany; 9 Institute of Biometry and Clinical Epidemiology, Charité – Universitätsmedizin Berlin, corporate member of Freie Universität Berlin and Humboldt-Universität zu Berlin, Berlin, Germany; 10 Childrens’ Hospital, School of Medicine, Technical University of Munich, Munich, Germany; 11 German Center for Infection Research (DZIF), Berlin, Germany; 12 AGV Research Unit Gene Vectors, Helmholtz Center Munich (HMGU), Munich, Germany

**Keywords:** Epidemiology, Fatigue, Neurological disorders

**Correction to**: *Nature Communications* 10.1038/s41467-022-32507-6, published online 30 August 2022

In the author list of this article, the names of the authors were incorrectly listed with initials and family name only. The incorrect author list read as “C. Kedor, H. Freitag, L. Meyer-Arndt, K. Wittke, L. G. Hanitsch, T. Zoller, F. Steinbeis, M. Haffke, G. Rudolf, B. Heidecker, T. Bobbert, J. Spranger, H. D. Volk, C. Skurk, F. Konietschke, F. Paul, U. Behrends, J. Bellmann-Strobl and C. Scheibenbogen”.

The author list has now been amended to include the given and family names in the HTML and PDF versions of the article. The corrected author list reads as “Claudia Kedor, Helma Freitag, Lil Meyer-Arndt, Kirsten Wittke, Leif G. Hanitsch, Thomas Zoller, Fridolin Steinbeis, Milan Haffke, Gordon Rudolf, Bettina Heidecker, Thomas Bobbert, Joachim Spranger, Hans-Dieter Volk, Carsten Skurk, Frank Konietschke, Friedemann Paul, Uta Behrends, Judith Bellmann-Strobl and Carmen Scheibenbogen”.

