# Supplementary material for: A prospective observational study of post-COVID-19 chronic fatigue syndrome following the first pandemic wave in Germany and biomarkers associated with symptom severity
Source: Nat Commun. 2022 Aug 30;13:5104. doi: 10.1038/s41467-022-32507-6 (PMC9426365; doi:10.1038/s41467-022-32507-6)
Supplement: Supplementary file 1 — Supplementary Information [file 41467_2022_32507_MOESM1_ESM.pdf]

**Supplementary Information for Post COVID-19 Chronic Fatigue Syndrome following the first pandemic wave in Germany and biomarkers associated with symptom severity results from a prospective observational study**

**List of Supplementary Information:**

**Figure S1:** Severity of stress intolerance and of hypersensitivity to temperature, light, and noise in PCS/ME/CFS and PCS/non-ME/CFS patients.
**Figure S2**: Consort flow diagram
**Table S1:** Ten most frequent initial symptoms of COVID-19 reported by patients
**Tables S2-S12**: Correlation analysis with standard error and test statistic for Tables 1-4 and Figures 1-4

**Supplementary Note 1: Protocol Synopsis**

| | |
|---|---|
| **Questionnaires:** | **CCC-Symptom Score** |
| | **PEM Questionnaire** |
| | **Chalder Fatigue Scale** |
| | **Bell disability scale** |
| | **COMPASS31** |
| **Assessments:** | **POTS** |
| | **Hand grip strength** |

**Figure S1:** Severity of stress intolerance and of hypersensitivity to temperature, light, and noise in PCS/ME/CFS and PCS/non-ME/CFS patients.

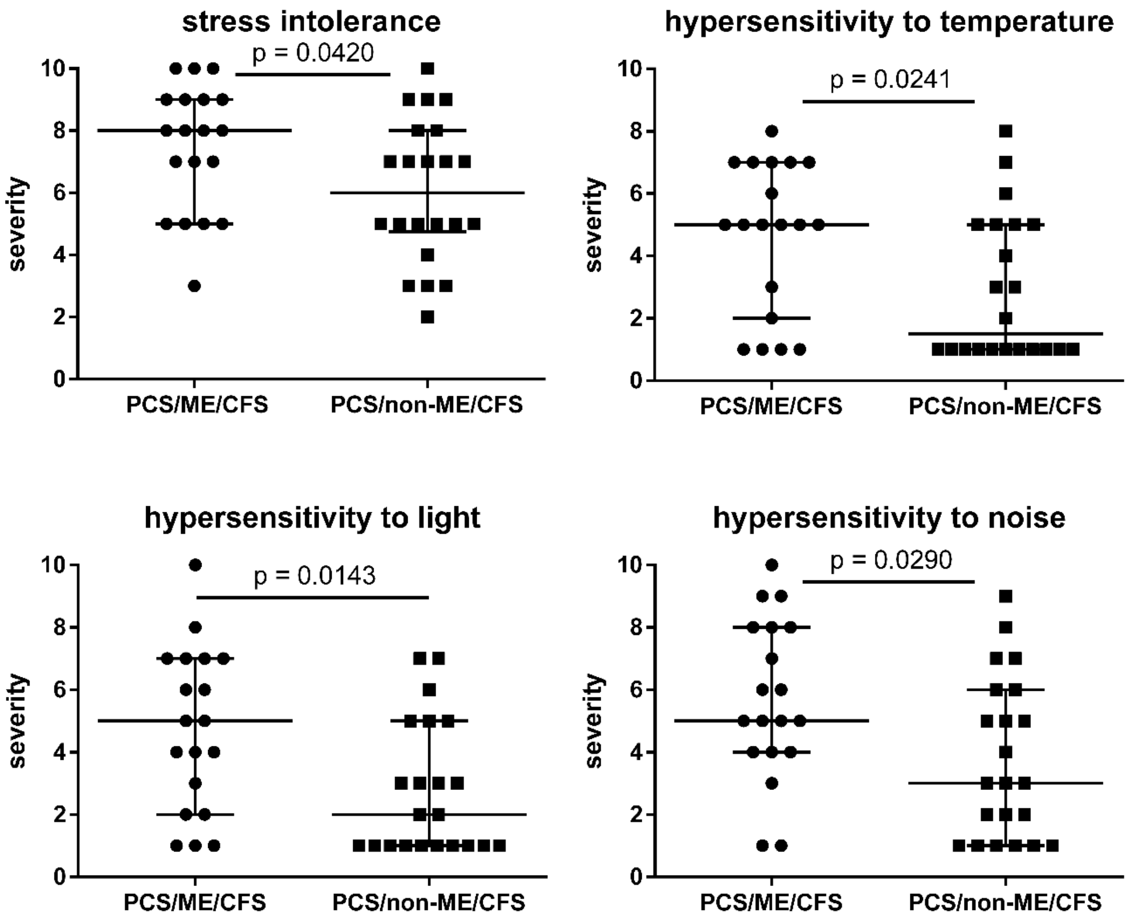


PCS/non-ME/CFS: post-COVID-19 syndrome/non-myalgic encephalomyelitis/chronic fatigue syndrome; PCS/ME/CFS: post-COVID-19 syndrome/myalgic encephalomyelitis/chronic fatigue syndrome.

There is a higher symptom burden for stress intolerance, and hypersensitivity to temperature, noise and light in the PCS/ME/CFS (n=19) compared to the PCS/non-ME/CFS (n=22) cohort. Median are shown with IQR (interquartile range). The samples are compared with (two-sided) Brunner-Munzel tests. We accounted for multiplicity with the BH-correction. Source data are provided as a Source Data file.

**Figure S2**: Consort flow diagram

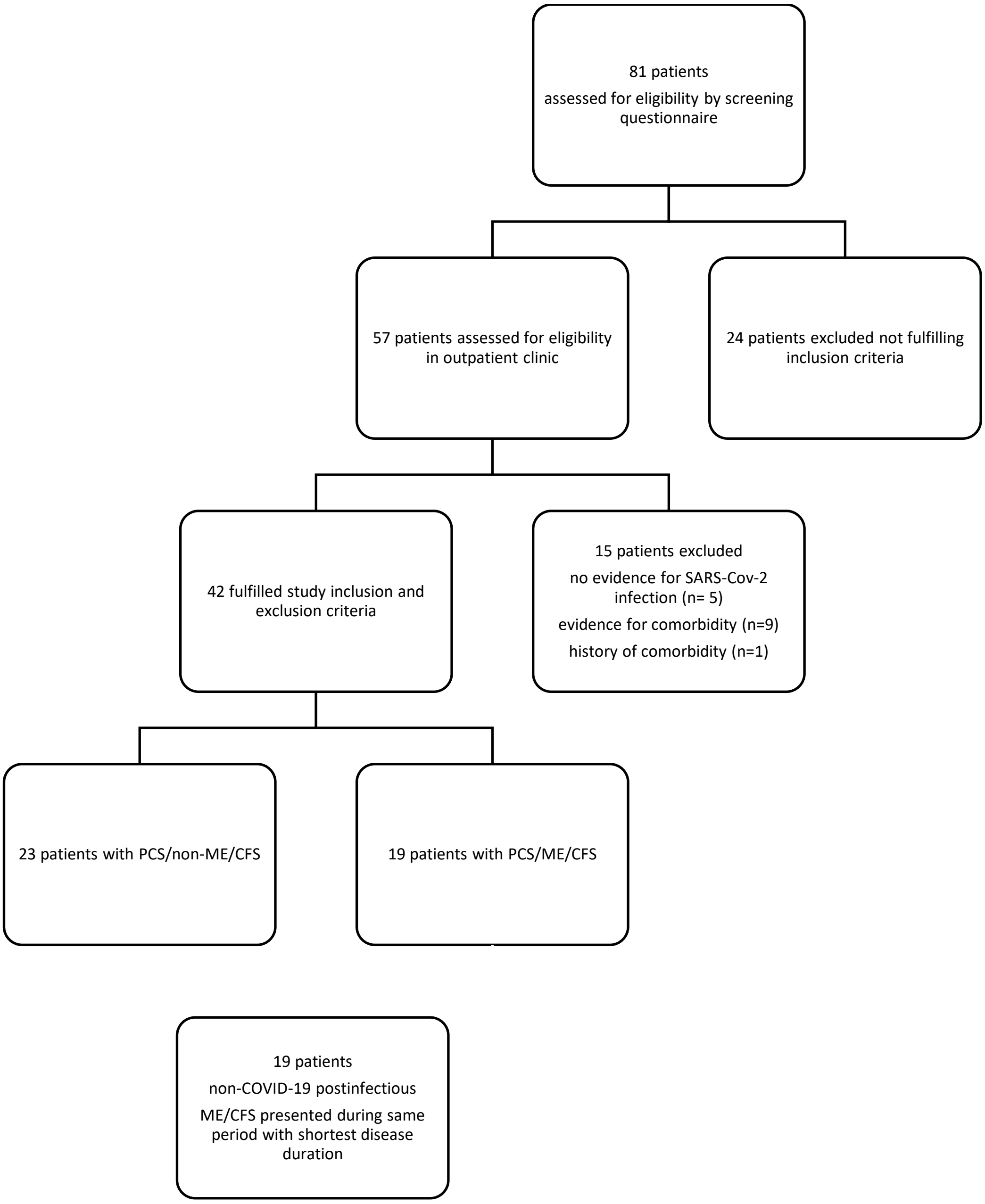


PCS/non-ME/CFS: post-COVID-19 syndrome/non-myalgic encephalomyelitis/chronic fatigue syndrome; PCS/ME/CFS: post-COVID-19 syndrome/myalgic encephalomyelitis/chronic fatigue syndrome.

All patients enrolled in this study presented at our outpatient clinics between August 2020 and November 2020. We informed patients on our website that our outpatient clinic offers a study for patients suffering from moderate to severe fatigue and exertion intolerance 3 to 6 months post COVID. Patients were selected for an appointment in our clinic based on a screening questionnaire, which specified our inclusion criteria. From a total of 81 patients, who contacted the Charité Fatigue Center during this time period, 24 were already excluded based on the screening questionnaire and 57 were assessed for eligibility in our outpatient clinic. From these, 42 fulfilled the inclusion criteria. 19 patients fulfilled the Canadian Consensus Criteria (CCC) for ME/CFS 1 (PCS/ME/CFS), the other 23 patients did not (PCS/non-ME/CFS).

From all postinfectious non-COVID-19 ME/CFS patients evaluated during the same period at our clinic (n=123) a sex- and age-matched control cohort who had the shortest duration of illness (13 months, range 7 - 19 months, n=19) was selected.

**Table S1.** Ten most frequent initial symptoms of COVID-19 reported by patients

| | PCS/ME/CFS | | | | PCS/non-ME/CFS | | | | all patients | |
|---|---|---|---|---|---|---|---|---|---|---|
| | male | female | total | % PCS/CFS | male | female | total | % PCS | total | % total |
| n | 5 | 14 | 19 | | 8 | 15 | 23 | | 42 | |
| Fatigue/malaise | 4 | 9 | **13** | 68 | 7 | 15 | **22** | 96 | **35** | 83 |
| Fever/ high temperature | 3 | 9 | **12** | 63 | 6 | 10 | **16** | 70 | **28** | 67 |
| Cough | 2 | 8 | **10** | 53 | 7 | 8 | **15** | 65 | **25** | 60 |
| Resp. problems | 2 | 8 | **10** | 53 | 5 | 9 | **14** | 61 | **24** | 57 |
| Loss of smell | 0 | 10 | **10** | 53 | 5 | 8 | **13** | 57 | **23** | 55 |
| Loss of taste | 0 | 10 | **10** | 53 | 4 | 8 | **12** | 52 | **22** | 52 |
| Headache | 2 | 9 | **11** | 58 | 2 | 9 | **11** | 48 | **22** | 52 |
| Arthralgia | 0 | 8 | **8** | 42 | 2 | 7 | **9** | 39 | **17** | 40 |
| Chest pain | 2 | 6 | **8** | 42 | 3 | 6 | **9** | 39 | **17** | 40 |
| Gastrointestinal symptoms | 1 | 8 | **9** | 47 | 1 | 6 | **7** | 30 | **16** | 38 |

This table shows the ten most frequent initial symptoms of COVID-19 reported by the 42 patients analysed in this study.

As additional information, we report the estimated effects, their standard errors, values of the test statistics and 95% simultaneous confidence intervals. The effect estimators are the contrasts (differences) of estimated relative effects (Wilcoxon-Mann-Whitney parameters).

**Table S2**: Effect estimators, standard errors, values of the test statistics, 95% simultaneous confidence intervals and degrees of freedom (DF) obtained from the nonparametric all-pairs Dunn-type multiple contrast tests (cf. Table 1, demographic data).

| | non-COVID ME/CFS vs PCS/non-ME/CFS | | | | | non-COVID ME/CFS vs PCS/ME/CFS | | | | | PCS/non-ME/CFS vs PCS/ME/CFS | | | | | |
|---|---|---|---|---|---|---|---|---|---|---|---|---|---|---|---|---|
| | Effect | Std. Error | T | Lower | Upper | Effect | Std. Error | T | Lower | Upper | Effect | Std. Error | T | Lower | Upper | DF |
| age | -0,1066 | 0,0923 | -1,1463 | -0,3232 | 0,1205 | 0,003 | 0,0958 | 0,0308 | -0,2271 | 0,2327 | 0,1096 | 0,0873 | 1,2457 | -0,1054 | 0,3148 | 36 |
| gender | -0,0423 | 0,0726 | -0,5824 | -0,216 | 0,134 | 0 | 0,0734 | 0 | -0,1768 | 0,1768 | 0,0423 | 0,0726 | 0,5824 | -0,134 | 0,216 | 39 |
| bmi | 0,0938 | 0,0883 | 1,0566 | -0,1229 | 0,302 | 0,1854 | 0,0898 | 2,0169 | -0,0396 | 0,3925 | 0,0915 | 0,0898 | 1,0139 | -0,1287 | 0,3032 | 36 |

**Table S3** Effect estimators of the relative effects, their standard errors, values of the test statistics and 95% confidence intervals obtained from the Brunner-Munzel tests (cf Table 1, PHQ9 and ESS in PCS).

| | PCS/non ME/CFS vs PCS/ME/CFS | | | | |
|---|---|---|---|---|---|
| | Effect | Std,Error | T | Lower | Upper |
| PHQ | 0,5824 | 0,0908 | 0,9075 | 0,3988 | 0,766 |
| ESS | 0,5172 | 0,0912 | 0,1882 | 0,3329 | 0,7015 |

**Table S4**: Effect estimators, standard errors, values of the test statistics, 95% simultaneous confidence intervals and degrees of freedom (DF) obtained from the nonparametric all-pairs Dunn-type multiple contrast tests (cf Table 2, symptom score)

| | non-COVID ME/CFS vs PCS/non-ME/CFS | | | | | non-COVID ME/CFS vs PCS/ME/CFS | | | | | PCS/non-ME/CFS vs PCS/ME/CFS | | | | | |
|---|---|---|---|---|---|---|---|---|---|---|---|---|---|---|---|---|
| | Effect | Std. Error | T | Lower | Upper | Effect | Std. Error | T | Lower | Upper | Effect | Std. Error | T | Lower | Upper | DF |
| sy_1 fatigue | -0,2897 | 0,0797 | -3,4262 | -0,4708 | -0,0851 | -0,2257 | 0,0763 | -2,8583 | -0,4022 | -0,0331 | 0,0639 | 0,0845 | 0,7544 | -0,1426 | 0,2651 | 35 |
| sy_2 PEM | -0,401 | 0,0596 | -5,9824 | -0,5359 | -0,2462 | -0,1914 | 0,0734 | -2,542 | -0,3627 | -0,0075 | 0,2096 | 0,0714 | 2,8489 | 0,0303 | 0,3759 | 35 |
| sy_3 need for rest | -0,2621 | 0,0823 | -3,036 | -0,4503 | -0,0515 | -0,1425 | 0,0824 | -1,707 | -0,3362 | 0,0626 | 0,1195 | 0,0862 | 1,374 | -0,094 | 0,3226 | 33 |
| sy_4 imp. performance | -0,2132 | 0,0816 | -2,5329 | -0,4019 | -0,0073 | -0,1278 | 0,0895 | -1,4121 | -0,3375 | 0,094 | 0,0854 | 0,0905 | 0,9392 | -0,1367 | 0,2994 | 34 |
| sy_5 stress intolerance | -0,3718 | 0,0633 | -5,317 | -0,5167 | -0,2062 | -0,1955 | 0,0772 | -2,4674 | -0,3768 | 1,00E-04 | 0,1763 | 0,0836 | 2,0649 | -0,0348 | 0,3723 | 28 |
| sy_6 muscle pain | -0,2115 | 0,0901 | -2,2763 | -0,4189 | 0,017 | -0,1747 | 0,0936 | -1,8282 | -0,3915 | 0,0605 | 0,0368 | 0,0858 | 0,4287 | -0,1725 | 0,243 | 32 |
| sy_7 headache | -0,2282 | 0,0798 | -2,7598 | -0,4127 | -0,0258 | -0,1596 | 0,091 | -1,7235 | -0,3714 | 0,0681 | 0,0687 | 0,0925 | 0,7404 | -0,1578 | 0,2883 | 32 |
| sy_8 joint pain | -0,0683 | 0,0975 | -0,6984 | -0,3001 | 0,1711 | 0,0024 | 0,1034 | 0,023 | -0,247 | 0,2515 | 0,0707 | 0,0893 | 0,7893 | -0,149 | 0,2837 | 30 |
| sy_9 memory | -0,0919 | 0,1027 | -0,8898 | -0,3363 | 0,1641 | -0,0598 | 0,0907 | -0,6574 | -0,2788 | 0,1652 | 0,0321 | 0,0921 | 0,3486 | -0,1947 | 0,2557 | 25 |
| sy_10 concentration | -0,2195 | 0,0902 | -2,3558 | -0,4271 | 0,01 | -0,1422 | 0,0843 | -1,6642 | -0,3407 | 0,0685 | 0,0773 | 0,0865 | 0,8905 | -0,1358 | 0,2836 | 31 |
| sy_11 mental fatigue | -0,2378 | 0,0807 | -2,8342 | -0,4235 | -0,0329 | -0,2034 | 0,0835 | -2,368 | -0,3966 | 0,007 | 0,0344 | 0,0902 | 0,3812 | -0,1846 | 0,2502 | 34 |
| sy_12 visual disturbances | -0,0147 | 0,0889 | -0,1657 | -0,229 | 0,2009 | 0,056 | 0,0929 | 0,6013 | -0,1711 | 0,2774 | 0,0707 | 0,0949 | 0,7426 | -0,1619 | 0,2959 | 32 |
| sy_15 palpitations | -0,0769 | 0,0948 | -0,808 | -0,3009 | 0,1552 | -0,007 | 0,0965 | -0,0729 | -0,2386 | 0,2253 | 0,0699 | 0,0923 | 0,7548 | -0,1557 | 0,2886 | 34 |
| sy_16 Dizziness when standing | -0,1489 | 0,0914 | -1,6054 | -0,3632 | 0,0803 | -0,0908 | 0,0998 | -0,9046 | -0,3269 | 0,156 | 0,0581 | 0,0881 | 0,6578 | -0,1586 | 0,2694 | 29 |
| sy_17 Dizziness when walking | -0,1967 | 0,0861 | -2,2254 | -0,3962 | 0,0204 | -0,1021 | 0,0952 | -1,0652 | -0,3261 | 0,1327 | 0,0946 | 0,0912 | 1,0317 | -0,13 | 0,31 | 33 |
| sy_18 sleep dist | -0,1188 | 0,0969 | -1,2146 | -0,3465 | 0,1221 | -0,1213 | 0,0971 | -1,2377 | -0,3493 | 0,1202 | -0,0025 | 0,0859 | -0,0292 | -0,2109 | 0,2061 | 30 |
| sy_19 hypersens. temp | -0,3629 | 0,0752 | -4,3908 | -0,5321 | -0,1658 | -0,189 | 0,0835 | -2,2089 | -0,3835 | 0,0216 | 0,1739 | 0,0737 | 2,3108 | -0,0112 | 0,3475 | 31 |
| sy_20 hypersens. light | -0,2752 | 0,0811 | -3,2177 | -0,4604 | -0,0671 | -0,0616 | 0,093 | -0,6607 | -0,2828 | 0,1658 | 0,2136 | 0,079 | 2,6192 | 0,0138 | 0,397 | 32 |
| sy_21 hypersens. noise | -0,2622 | 0,0853 | -2,931 | -0,4569 | -0,0436 | -0,0758 | 0,0933 | -0,8093 | -0,297 | 0,1532 | 0,1865 | 0,0811 | 2,2465 | -0,0175 | 0,3755 | 32 |
| sy_22 breathing disorder | 0,1992 | 0,0819 | 2,3665 | -0,0073 | 0,3893 | 0,2416 | 0,0832 | 2,7884 | 0,0297 | 0,4326 | 0,0424 | 0,0896 | 0,4726 | -0,1759 | 0,2567 | 33 |
| sy_23 Irritable bowl | -0,1972 | 0,0938 | -2,0473 | -0,4137 | 0,0404 | -0,0866 | 0,0907 | -0,9502 | -0,3019 | 0,1372 | 0,1106 | 0,0883 | 1,2419 | -0,1086 | 0,3196 | 31 |
| sy_24 fever | -0,0837 | 0,0813 | -1,0247 | -0,2814 | 0,1208 | -0,0716 | 0,0877 | -0,8129 | -0,2847 | 0,1483 | 0,0122 | 0,0613 | 0,1983 | -0,1405 | 0,1643 | 21 |
| sy_25 painfull ymphnodes | -0,245 | 0,0788 | -2,9825 | -0,427 | -0,0439 | -0,2361 | 0,0836 | -2,7195 | -0,4286 | -0,0231 | 0,0089 | 0,0744 | 0,1198 | -0,1723 | 0,1895 | 32 |
| sy_26 sorethroat | -0,286 | 0,0846 | -3,1947 | -0,4783 | -0,0675 | -0,2302 | 0,0863 | -2,573 | -0,429 | -0,0102 | 0,0558 | 0,0788 | 0,7061 | -0,1379 | 0,2453 | 31 |
| sy_27 flu-like symptoms | -0,3815 | 0,0706 | -4,8596 | -0,54 | -0,1968 | -0,2541 | 0,0679 | -3,5801 | -0,4115 | -0,082 | 0,1274 | 0,0862 | 1,4614 | -0,0863 | 0,3298 | 32 |

**Table S5**: Effect estimators, standard errors, values of the test statistics, 95% simultaneous confidence intervals and degrees of freedom obtained from the nonparametric all-pairs Dunn-type multiple contrast tests (cf Table 3, autonomic dysfunction – COMPASS31)

| | non-COVID ME/CFS vs PCS/non-ME/CFS | | | | | non-COVID ME/CFS vs PCS/ME/CFS | | | | | PCS/non-ME/CFS vs PCS/ME/CFS | | | | | |
|---|---|---|---|---|---|---|---|---|---|---|---|---|---|---|---|---|
| | Effect | Std. Error | T | Lower | Upper | Effect | Std. Error | T | Lower | Upper | Effect | Std. Error | T | Lower | Upper | DF |
| com_total | -0,2834 | 0,0781 | -3,4326 | -0,4618 | -0,0829 | -0,1068 | 0,0942 | -1,1251 | -0,3283 | 0,1259 | 0,1766 | 0,0839 | 2,0609 | -0,034 | 0,3721 | 32 |
| com_orth | -0,2164 | 0,0828 | -2,5321 | -0,4071 | -0,0077 | -0,053 | 0,0927 | -0,5702 | -0,2731 | 0,1724 | 0,1635 | 0,0853 | 1,8829 | -0,0491 | 0,3618 | 36 |
| com_vaso | -0,082 | 0,061 | -1,3389 | -0,2286 | 0,0682 | 8,00E-04 | 0,073 | 0,0104 | -0,1764 | 0,1779 | 0,0828 | 0,0613 | 1,3445 | -0,0682 | 0,23 | 32 |
| com_secr | -0,1633 | 0,0835 | -1,9213 | -0,3582 | 0,0452 | -0,0589 | 0,0967 | -0,6079 | -0,288 | 0,1766 | 0,1044 | 0,0879 | 1,1798 | -0,1122 | 0,3116 | 34 |
| com_gi | -0,102 | 0,0856 | -1,1835 | -0,3049 | 0,1096 | -0,132 | 0,0911 | -1,4317 | -0,3456 | 0,0947 | -0,0299 | 0,0963 | -0,3107 | -0,2606 | 0,2039 | 32 |
| com_blad | -0,003 | 0,0774 | -0,0389 | -0,1918 | 0,186 | 0,0683 | 0,0901 | 0,7563 | -0,1538 | 0,2839 | 0,0714 | 0,0907 | 0,7843 | -0,1523 | 0,2881 | 29 |
| com_pupil | -0,1292 | 0,0894 | -1,4295 | -0,3381 | 0,0919 | -0,0444 | 0,0974 | -0,4557 | -0,2755 | 0,1915 | 0,0848 | 0,0892 | 0,9458 | -0,1337 | 0,2954 | 36 |

**Table S6**: Standard errors of the correlation estimates of Spearman's ρ and associated test statistics (cf. Table 4) (cf Table 4, laboratory values and HGS)

| | | Standard Error | | | | Test Statistic | | | |
|---|---|---|---|---|---|---|---|---|---|
| | | Fmax1 | Fmean1 | Fmax2 | Fmean2 | Fmax1 | Fmean1 | Fmax2 | Fmean2 |
| Hb | PCS/non-ME/CFS | 0,2033 | 0,1885 | 0,1851 | 0,1914 | 2,2795 | 3,0256 | 3,1937 | 2,8808 |
| Ferritin | PCS/non-ME/CFS | 0,2297 | 0,2319 | 0,234 | 0,2342 | 0,9792 | 0,7717 | 0,5076 | 0,4816 |
| Bilirubin | PCS/non-ME/CFS | 0,2357 | 0,2353 | 0,2351 | 0,2354 | -0,0862 | 0,2397 | 0,3007 | 0,2269 |
| Kreatinin | PCS/non-ME/CFS | 0,2066 | 0,207 | 0,2147 | 0,2153 | 2,3276 | 2,3123 | 1,9203 | 1,8908 |
| CRP | PCS/non-ME/CFS | 0,1965 | 0,2071 | 0,1999 | 0,1931 | -2,6255 | -2,0787 | -2,4547 | -2,7983 |
| IL8 | PCS/non-ME/CFS | 0,1895 | 0,2063 | 0,2039 | 0,2019 | -2,9721 | -2,1187 | -2,2494 | -2,3518 |
| NTproBNP | PCS/non-ME/CFS | 0,2313 | 0,2316 | 0,2269 | 0,2283 | -0,8325 | -0,7985 | -1,1929 | -1,0867 |
| ACE1 | PCS/non-ME/CFS | 0,2295 | 0,2335 | 0,23 | 0,2325 | -0,9896 | -0,5795 | -0,9479 | -0,7116 |
| ACE2 | PCS/non-ME/CFS | 0,2366 | 0,2201 | 0,2114 | 0,2037 | 1,3668 | 2,1543 | 2,527 | 2,8446 |
| Hb | PCS/ME/CFS | 0,1861 | 0,1589 | 0,1777 | 0,174 | 3,4448 | 4,7546 | 3,9603 | 4,1269 |
| Ferritin | PCS/ME/CFS | 0,2251 | 0,2044 | 0,2088 | 0,2084 | 1,652 | 2,6356 | 2,6346 | 2,6488 |
| Bilirubin | PCS/ME/CFS | 0,2141 | 0,212 | 0,2315 | 0,2245 | 2,4112 | 2,4979 | 1,912 | 2,2015 |
| Kreatinin | PCS/ME/CFS | 0,234 | 0,2224 | 0,2209 | 0,2248 | 1,5056 | 2,053 | 2,3441 | 2,1889 |
| CRP | PCS/ME/CFS | 0,2417 | 0,2425 | 0,2484 | 0,2495 | -0,347 | -0,0446 | 0,4497 | 0,2492 |
| IL8 | PCS/ME/CFS | 0,2351 | 0,2412 | 0,2494 | 0,2495 | -1,0447 | -0,4364 | -0,2856 | -0,2523 |
| NTproBNP | PCS/ME/CFS | 0,1779 | 0,1641 | 0,1548 | 0,1617 | -3,9516 | -4,5969 | -5,0745 | -4,7162 |
| ACE1 | PCS/ME/CFS | 0,2472 | 0,2513 | 0,2514 | 0,2523 | -1,1701 | -0,917 | -0,9067 | -0,8453 |
| ACE2 | PCS/ME/CFS | 0,2464 | 0,2454 | 0,254 | 0,2541 | -0,6833 | -0,7788 | -0,7102 | -0,6952 |
| Hb | non-COVID ME/CFS | 0,2756 | 0,2746 | 0,2732 | 0,2717 | 0,4101 | 0,5095 | 0,6303 | 0,7394 |
| Ferritin | non-COVID ME/CFS | 0,2999 | 0,2945 | 0,2974 | 0,2921 | -0,3481 | -0,7276 | -0,5543 | -0,8463 |
| Bilirubin | non-COVID ME/CFS | 0,2489 | 0,2454 | 0,2387 | 0,2557 | 1,7709 | 1,8987 | 2,1319 | 1,5137 |
| Kreatinin | non-COVID ME/CFS | 0,2766 | 0,2746 | 0,2717 | 0,2695 | 0,2654 | 0,5152 | 0,738 | 0,877 |
| CRP | non-COVID ME/CFS | 0,2772 | 0,2764 | 0,2764 | 0,275 | 0,1232 | 0,2991 | 0,3056 | 0,4771 |
| IL8 | non-COVID ME/CFS | 0,2389 | 0,2004 | 0,235 | 0,2389 | -2,5524 | -3,7294 | -2,665 | -2,5524 |
| NTproBNP | non-COVID ME/CFS | 0,2639 | 0,2594 | 0,2588 | 0,2592 | -1,1648 | -1,3642 | -1,3879 | -1,3721 |
| ACE1 | non-COVID ME/CFS | 0,2771 | 0,2758 | 0,2773 | 0,2763 | -0,1418 | -0,3885 | -0,0129 | -0,3102 |

**Table S7**: Standard errors of the correlation estimates of Spearman's ρ and associated test statistics.

| | | Standard Error | | | | Test statistic | | | |
|---|---|---|---|---|---|---|---|---|---|
| | | CFQ | CFQA | CFQB | Fatigue | CFQ | CFQA | CFQB | Fatigue |
| fT3 | PCS/non-ME/CFS | 0,218 | 0,2174 | 0,2091 | 0,2167 | 0,2078 | -0,3875 | 1,3661 | -0,5431 |
| fT4 | PCS/non-ME/CFS | 0,2122 | 0,2157 | 0,2102 | 0,1945 | -1,0973 | -0,697 | -1,2779 | -2,3335 |
| TSH | PCS/non-ME/CFS | 0,2255 | 0,2292 | 0,213 | 0,2257 | 0,8107 | 0,1687 | 1,7438 | 0,7994 |
| Thromb | PCS/non-ME/CFS | 0,2181 | 0,2182 | 0,2182 | 0,2157 | -0,1653 | -0,016 | 0,0629 | 0,7068 |
| fT3 | PCS/ME/CFS | 0,2034 | 0,2486 | 0,2151 | 0,2471 | -2,8574 | -0,4314 | -2,3702 | -0,615 |
| fT4 | PCS/ME/CFS | 0,2488 | 0,2495 | 0,2435 | 0,2453 | -0,3896 | 0,2423 | -0,9332 | -0,7904 |
| TSH | PCS/ME/CFS | 0,2271 | 0,25 | 0,2105 | 0,2499 | -1,8402 | 0,0272 | -2,5615 | 0,1199 |
| Thromb | PCS/ME/CFS | 0,2415 | 0,2254 | 0,2407 | 0,1768 | -0,3761 | -1,6381 | 0,5084 | -3,8726 |

**Table S8**: Effect estimators, standard errors, values of the test statistics, 95% simultaneous confidence intervals and degrees of freedom obtained from the nonparametric all-pairs Dunn-type multiple contrast tests (cf. Figure 1, Bell scale, Chader Fatigue Scale and SF36 physical functioning)

| | non-COVID ME/CFS vs PCS/non-ME/CFS | | | | | non-COVID ME/CFS vs PCS/ME/CFS | | | | | PCS/non-ME/CFS vs PCS/ME/CFS | | | | | |
|---|---|---|---|---|---|---|---|---|---|---|---|---|---|---|---|---|
| | Effect | Std. Error | T | Lower | Upper | Effect | Std. Error | T | Lower | Upper | Effect | Std. Error | T | Lower | Upper | DF |
| Bell | 0,2704 | 0,0703 | 3,6577 | 0,0906 | 0,4332 | 0,1341 | 0,0864 | 1,5336 | -0,0812 | 0,3374 | -0,1364 | 0,0886 | -1,5192 | -0,3447 | 0,0847 | 30 |
| CFQ | -0,331 | 0,0732 | -4,1868 | -0,4965 | -0,1423 | -0,1037 | 0,08 | -1,2867 | -0,2927 | 0,0932 | 0,2273 | 0,079 | 2,7775 | 0,0279 | 0,4094 | 36 |
| SF36 physical | 0,2316 | 0,0816 | 2,7374 | 0,025 | 0,4193 | 0,1854 | 0,0928 | 1,951 | -0,0477 | 0,3993 | -0,0463 | 0,0897 | -0,515 | -0,2602 | 0,172 | 34 |

**Table S9:** Effect estimators of the relative effects, their standard errors, values of the test statistics and 95% confidence intervals obtained from the Brunner-Munzel tests (cf Figure 1, SF36 role limitation, energy and social)

| | PCS vs PCS/ME/CFS | | | | |
|---|---|---|---|---|---|
| | Effect | Std. Error | T | Lower | Upper |
| SF36 role limitations | 0,3261 | 0,0508 | -3,4254 | 0,2208 | 0,4314 |
| SF36 energy | 0,3318 | 0,0825 | -2,0377 | 0,165 | 0,4986 |
| SF36 social | 0,3389 | 0,0883 | -1,8241 | 0,1595 | 0,5183 |

**Table S10**: Effect estimators, standard errors, values of the test statistics, 95% simultaneous confidence intervals and degrees of freedom obtained from the nonparametric all-pairs Dunn-type multiple contrast tests (cf. Figure 2, PEM frequency, severity and length)

| | non-COVID ME/CFS vs PCS/non-ME/CFS | | | | | non-COVID ME/CFS vs PCS/ME/CFS | | | | | PCS/non-ME/CFS vs PCS/ME/CFS | | | | | |
|---|---|---|---|---|---|---|---|---|---|---|---|---|---|---|---|---|
| | Effect | Std. Error | T | Lower | Upper | Effect | Std. Error | T | Lower | Upper | Effect | Std. Error | T | Lower | Upper | DF |
| PEM_frequency | -0,3281 | 0,077 | -3,9468 | -0,5033 | -0,127 | -0,1279 | 0,0916 | -1,3817 | -0,3437 | 0,1008 | 0,2002 | 0,076 | 2,5615 | 0,0074 | 0,3786 | 28 |
| PEM_severity | -0,2715 | 0,0736 | -3,5076 | -0,4414 | -0,0829 | -0,1426 | 0,0898 | -1,5676 | -0,3533 | 0,0817 | 0,1289 | 0,0914 | 1,3942 | -0,0989 | 0,3439 | 30 |
| PEM_length | -0,4299 | 0,0635 | -5,9004 | -0,5721 | -0,2625 | -0,0895 | 0,0641 | -1,389 | -0,2431 | 0,0685 | 0,3404 | 0,0664 | 4,7196 | 0,1688 | 0,492 | 34 |

**Table S11**: Effect estimators, standard errors, values of the test statistics, 95% simultaneous confidence intervals and degrees of freedom obtained from the nonparametric all-pairs Dunn-type multiple contrast tests (cf Figure 3, HGS)

| | non-COVID ME/CFS vs PCS/non-ME/CFS | | | | | non-COVID ME/CFS vs PCS/ME/CFS | | | | | PCS/non-COVID ME/CFS vs PCS/ME/CFS | | | | | |
|---|---|---|---|---|---|---|---|---|---|---|---|---|---|---|---|---|
| | Effect | Std. Error | T | Lower | Upper | Effect | Std. Error | T | Lower | Upper | Effect | Std. Error | T | Lower | Upper | DF |
| Fmax1 | 0,1592 | 0,1046 | 1,4963 | -0,1111 | 0,4076 | -0,0303 | 0,1235 | -0,245 | -0,3309 | 0,2759 | -0,1895 | 0,1065 | -1,7361 | -0,4398 | 0,0881 | 19 |
| Fmax2 | 0,1716 | 0,0988 | 1,7018 | -0,0848 | 0,4067 | -0,0621 | 0,1204 | -0,5145 | -0,3531 | 0,2398 | -0,2337 | 0,1124 | -2,0021 | -0,4929 | 0,0635 | 19 |
| Fmean1 | 0,1695 | 0,1021 | 1,6283 | -0,0916 | 0,4087 | -0,0664 | 0,1156 | -0,5722 | -0,3426 | 0,2204 | -0,2358 | 0,1058 | -2,1451 | -0,4783 | 0,04 | 23 |
| Fmean2 | 0,1834 | 0,0955 | 1,8776 | -0,0633 | 0,409 | -0,1006 | 0,1149 | -0,8696 | -0,3742 | 0,1891 | -0,284 | 0,1126 | -2,3855 | -0,5373 | 0,0163 | 21 |

**Table S12**: Effect estimators of the relative effects, their standard errors, values of the test statistics and 95% confidence intervals obtained from the Brunner-Munzel tests (cf Figure 4, Pulse and arterial pressure)

| | | p. hat | S.E | T | Lower | Upper |
|---|---|---|---|---|---|---|
| PCS/non-ME/CFS | Systole | 0,538 | 0,179 | 0,822 | 0,439 | 0,636 |
| PCS/non-ME/CFS | Diastole | 0,727 | 0,3038 | 2,894 | 0,559 | 0,895 |
| PCS/non-ME/CFS | Puls | 0,758 | 0,1805 | 5,536 | 0,658 | 0,858 |
| PCS/ME/CFS | Systole | 0,429 | 0,1718 | -1,601 | 0,332 | 0,525 |
| PCS/ME/CFS | Diastole | 0,546 | 0,1984 | 0,898 | 0,435 | 0,656 |
| PCS/ME/CFS | Puls | 0,804 | 0,2955 | 3,985 | 0,639 | 0,968 |

## Supplementary Note 1: Study Protocol

# STUDY REGISTRATION FORM

**1. Registration Date** 22.07.2020 (dd.mm.yyyy)

**2. Principal Investigator**

| | |
|---|---|
| Title | Prof. Dr. med. |
| First Name | Carmen |
| Surname | Scheibenbogen |
| Institute | Institut für Med. Immunologie, CVK |
| Research Group | AG Scheibenbogen |
| E-Mail address | carmen.scheibenbogen@charite.de |

**3. Co-Investigator (internal and/or external cooperation partners)**

(Please provide: Title, Name, Email, Address, Organisation)

Dr. med. Claudia Kedor
claudia.kedor@charite.de
Institut für Med. Immunologie, CVK

Dr. rer. medic. Helma Freitag
helma.freitag@charite.de
Institut für Med. Immunologie, CVK

**4. Is industry involved?** (If Yes, please provide name of cooperation or contract partners)

- (●) No
- ( ) Yes

**5. Title of Project or Proposal?**

Post-COVID-19 Chronic Fatigue Syndrom

## 6. Description of Project or Proposal

Please include rationale, central goals, main methods and impact for patients if applicable.(1.500 characters, including spaces)

Post-infectious chronic fatigue syndrome (CFS) occurs both epidemically and sporadically after infectious diseases, and this is well documented after the SARS pandemic of 2003. It is a complex, severe disease with a prevalence of 0.2 - 0.5%. Possible pathomechanisms are autoantibodies, autonomous and endothelial dysfunction and consecutive disturbed muscle metabolism.
First reports are already available from younger patients who report persistent fatigue and exercise intolerance more than 3 months after Covid-19 infection. Early characterization and treatment of post-Covid-19 syndromes is of high relevance to prevent chronification.
Patients who suffer from persistent fatigue and the cardinal symptoms of CFS (exertion/stress intolerance, cognitive disturbance according to IOM) 6 months after COVID-19 will be diagnosed in our outpatient clinic. As part of the diagnostic process, a hand strength test for muscular fatigue and a Schellong test for orthostatic intolerance are performed. Autoantibodies and soluble markers as well as gene variants associated with CFS are determined as biomarkers.

## 7. What type of research are you planning or conducting?

Epidemiologic work

## 8. If you plan a clinical study, did it already gain appropriate official approval?

- ○ No
- ◉ Yes, Charité Ethics Committee
- ○ Not applicable, because

## 9. What is the main goal of the project?

(Multiple choice possible)

- ☑ Diagnosis / Prediction / Stratification
- ☐ New Therapy
- ☐ Repurposed Therapy
- ☐ Vaccination
- ☐ Epidemiology
- ☑ Immunology
- ☐ Pathology
- ☑ Pathogenesis and Mechanism
- ☐ Digital Health
- ☐ Others

## 10. If patients are involved, is an interventional study planned?

(Multiple choice possible)

- ☐ Yes, small molecule/biologics
- ☐ Yes, ATMPs
- ☐ Yes, medical device or combinatory approach
- ☐ Yes, non-drug and non-medical device intervention
- ☑ No intervention

**11. Please describe the kind of intervention.** (max. 1.500 characters, including spaces)

| |
|---|
| |

**12. Which patient material is used?**
(Multiple choice possible)

- [x] Serum
- [ ] Plasma
- [x] Blood
- [ ] Smears
- [ ] Stool
- [ ] Urine
- [ ] CSF
- [ ] Biopsies
- [ ] Autopsies
- [ ] BAL
- [ ] Tissue
- [ ] Others

**13. If you plan a preclinical laboratory study, are there animals involved?**

- ○ Yes
- ◉ No

**14. What is the status of your project?**

Project running

**15. When does/did the project start?**

01.08.2020 (dd.mm.yyyy)

**16. What is the estimated duration of the project?**

6 (in months)

**17. Funding of ongoing project**

(Multiple choice possible)

- [x] Third Party
- [x] Intramural Funding (e. g. LOM)
- [ ] BIH
- [ ] BUA

**18. If applicable, what is the total funding volume (intramural, third party) of your (planned) project.**

| € 20.000 | (in Euro) |
|---|---|

**19. Has a manuscript of this project already been submitted for publication?**

- ○ Yes
- ◉ No

**20. What do you still need to implement your project?** (e.g. cooperation partner, technologies, access to research infrastructure (Core Facilities, Biosafety level laboratory ...)) (1000 characters, including spaces)

access to BIH infrastructure for data collection (Redcap) is initiated

**21. General comments or suggestions** (500 characters, including spaces)

**22. Do you agree that general information about your project** (your name, project title and project description) **will be made public on Charité's external and internal webpage outside the registry?**

- ◉ Yes
- ○ No

**CSC-Number** (Provided and entered by CSC!)

**Synopsis Post-Covid ME/CFS**
**Sub-study PA-Covid-19 EA2_066-20 Charité**

**Date V1 20.7.20 V2 21.1.21 V3 24.09.2021**

| | |
|---|---|
| **Coordinating team** | Carmen Scheibenbogen, Claudia Kedor, Helma Freitag, Silvia Thiel<br>Institute for Medical Immunology, Charité CVK |
| **Study design and plan** | Prospective observational study of patients with post COVID-19 fatigue and suspicion of ME/CFS |
| **Background** | Myalgic encephalomyelitis/chronic fatigue syndrome (ME/CFS) is a complex chronic disease with fatigue and post-exertional malaise (PEM) as cardinal symptoms. ME/CFS is often triggered by an infection, e.g. Epstein-Barr virus. After the SARS-CoV-1 epidemic in 2003, 27-40% of patients developed ME/CFS or similar symptoms as a result of the SARS-CoV-1 infection. Now, case reports of ME/CFS-typical long-term sequelae in the current pandemic of SARS-CoV-2 are also accumulating.<br>The aim of this observational study is to characterise post-COVID-19 ME/CFS, to investigate the pathomechanism as basis to develop biomarkers and intervention strategies.<br>Patients suffering from persistent fatigue and the cardinal symptoms of ME/CFS 6 months after COVID-19 (exercise intolerance, non-restorative sleep, cognitive disturbances according to IOM criteria) present to our outpatient clinic for diagnosis. The clinical data and validated questionnaires (Chalder Fatigue Score, SF36, Canadian Criteria weighted, Bell Score, COMPASS-31, ESS and PHQ9) collected during the diagnostic process, as well as routine laboratory values, are stored in REDCap.<br>As part of the diagnostic work-up, a hand strength test is performed for muscular fatigue and a Schellong test for orthostatic dysregulation. Autoantibodies and soluble markers as well as gene variants associated with ME/CFS will be determined as biomarkers. For the assessment of biomarkers the vote EA2/067/20 is available. |
| **Schedule of visits and assessments** | **Month 6**<br>- Routine laboratory Panel<br>- Canadian Consensus Criteria<br>- Canadian Criteria Scoring<br>- Chalder Fatigue Scale<br>- Bell Performance Status<br>- SF-36 |

| | |
|---|---|
| | - PHQ9<br>- ESS<br>- COMPASS-31<br>- Muscle Fatigue: Hand Strength<br>- Postural blood pressure and pulse<br>- ECG, ECHO, if POTS further cardiologic assessment<br>- Pulmonary function incl. O2 diffusion, chest X-ray or CT scan<br>- Neurology examination in case of severe headache and severe cognitive impairment<br>- Blood sampling for biomarkers<br>- optional: Endopat, HRV<br>**Month 9 and 18**<br>- Follow up questionnaires via REDCap<br>**Month 12 and 24**<br>- Follow up questionnaires via REDCap<br>- Muscle fatigue: hand strength<br>- Postural blood pressure and pulse<br>- Blood sampling for biomarkers |
| **Criteria for inclusion** | Patients suffering from persistent fatigue and cardinal symptoms of ME/CFS 6 months after COVID-19:<br>- Fatigue (moderate to severe)<br>- Cognitive disturbance<br>- Exercise intolerance with increase in symptoms >2 hours<br>- Pain (head or muscle)<br>- Sleep not restful<br>- Age: ≥ 18 years, ≤ 60 years<br>- evidence of COVID-19 (PCR, serology or typical clinic with sensory disturbances or CT) |
| **Criteria for exclusion** | - organ dysfunction due to COVID-19 (pulmonology, cardiology, neurology, laboratory)<br>- Major depression or anxiety disorder<br>- Sleep disorder<br>- Severe breathing difficulties<br>- Severe COVID-19 or O2 administration<br>- Pre-existing fatigue or disease that may be associated with chronic fatigue |

# CCC-Symptom Score

Date:
Name:

Skale 1-10
1= no Symptoms,…5= moderate Symptoms,…10= extreme Symptoms

| | Scale 1-10 | |
|---|---|---|
| FATIGUE | | |
| Exhaustion | | |
| Stress-dependent increase in | | |
| Fatigue/exhaustion the next day | | |
| Need for rest periods | | |
| Performance limitations in everyday life | | |
| Stress is not tolerated | | |
| PAIN | | |
| Muscle pain | | |
| Headache (new, new type, new intensity) | | |
| Joint pain | | |
| COGNITIVE PERFORMANCE | | |
| Memory/word finding disorders | | |
| Konzentrationsstörungen | | |
| Concentration disorders | | |
| Visual disturbances | | |
| Mood swings | | |
| Reading receptivity | | |
| CARDIOVASCULAR-CIRCULATION | | |
| Palpitations | | |
| Dizziness when standing up | | |
| Dizziness when walking | | |
| Further Symptoms | | |
| Sleep disturbance | | |
| Temperature sensitivity | | |
| Sensitivity to light | | |
| Sensitivity to noise | | |
| Breathing disorders | | |
| Irritable bowel with flatulence and/or diarrhea | | |
| Fever | | |
| Painful lymph nodes | | |
| Sore throat | | |
| Flu-like feeling | | |

## PEM Questionnaire [2]

For each symptom below, please indicate number for frequency and one number for severity: Please complete the chart from left to right.

| **Symptoms** | **Frequency:**<br>Throughout the past 6 months,<br>how often have you had this symptom?<br>For each symptom listed below, circle a number from:<br>0 = none of the time<br>1 = a little of the time<br>2 = about half the time<br>3 = most of the time<br>4 = all of the time | **Severity:**<br>Throughout the past 6 months,<br>how much has this symptom bothered you?<br>For each symptom listed below, circle a number from:<br>0 = symptom not present<br>1 = mild<br>2 = moderate<br>3 = severe<br>4= very severe |
|---|---|---|
| Dead, heavy feeling after starting to exercise | | |
| Next day soreness or fatigue after non-strenuous, everyday activities | | |
| Mentally tired after the slightest effort | | |
| Minimum exercise makes you physically tired | | |
| Physically drained or sick after mild activity | | |

For each question below, choose the answer which best describes your PEM symptoms.

<table>
<tr><td colspan="6">If you were to become exhausted after actively participating in extracurricular activities, sports, or outings with friends, would you recover within an hour or two after the activity ended?</td><td>yes</td><td>no</td></tr>
<tr><td colspan="6">If you were to become exhausted after actively participating in extracurricular activities, sports, or outings with friends, would you recover within an hour or two after the activity ended?</td><td>yes</td><td>no</td></tr>
<tr><td colspan="6">Do you experience a worsening of your fatigue/energy related illness after engaging in minimal physical effort?</td><td>yes</td><td>no</td></tr>
<tr><td colspan="6">Do you experience a worsening of your fatigue/energy related illness after engaging in mental effort?</td><td>yes</td><td>no</td></tr>
<tr><td>If you feel worse after activities, how long does this last?</td><td>≤ 1 h</td><td>2-3h</td><td>4-10h</td><td>11-13h</td><td>14-24h</td><td>≥24h</td><td>2-3 days</td></tr>
<tr><td colspan="6">If you do not exercise, is it because exercise makes your symptoms worse?</td><td>yes</td><td>no</td></tr>
</table>

# Chalder Fatigue Scale (CFQ) [3]

We would like to know more about any problems you have with feeling tired, weak or lack in energy in the last month. Please answer ALL the questions by ticking the answer which applies to you most closely. If you have been feeling tired for a long while, then compare yourself to how you felt when you were last well. Please tick only ONE box per line.

| | less than usual | not more than usual | more than usual | much more than usual |
|---|---|---|---|---|
| **1. Do you have problems with tiredness?** | **0** | **1** | **2** | **3** |
| **2. Do you need to rest more?** | **0** | **1** | **2** | **3** |
| **3. Do you feel sleepy or drowsy?** | **0** | **1** | **2** | **3** |
| **4. Do you have problems starting things?** | **0** | **1** | **2** | **3** |
| **5. Do you lack energy?** | **0** | **1** | **2** | **3** |
| **6. Do you have less strength in your muscles?** | **0** | **1** | **2** | **3** |
| **7. Do you feel weak?** | **0** | **1** | **2** | **3** |
| **8. Do you Have difficulties concentrating?** | **0** | **1** | **2** | **3** |
| **9. Do you make slips of the tongue when speaking?** | **0** | **1** | **2** | **3** |
| **10. Do you find it more difficult to find the right word?** | **0** | **1** | **2** | **3** |

| | better than usual | no worse than usual | worse than usual | much worse than usual |
|---|---|---|---|---|
| **11. How is your memory?** | **0** | **1** | **2** | **3** |

**Total score : __________**

**Scoring: a value between 0 and 33 is possible. The higher the score, the higher the fatigue.**

## Bell Disability Scale (ME/CFS) [4]

**100**: No symptoms with exercise. Normal overall activity. Able to work or do house/home work full time with no difficulty.

**90**: No symptoms at rest. Mild symptoms with physical activity. Normal overall activity level. Able to work full time without difficulty.

**80**: Mild symptoms at rest. Symptoms worsened by exertion. Minimal activity restriction needed for activities requiring exertion only. Able to work full time with difficulty in jobs requiring exertion.

**70**: Mild symptoms at rest. Some daily activity limitation clearly noted. Overall functioning close to 90% of expected except for activities requiring exertion. Able to work/do housework full time with difficulty. Needs to rest in day.

**60**: Mild to moderate symptoms at rest. Daily activity limitation clearly noted. Overall functioning 70% to 90%. Unable to work full time in jobs requiring physical labour (including just standing), but able to work full time in light activity (sitting) if hours are flexible.

**50**: Moderate symptoms at rest. Moderate to severe symptoms with exercise or activity; overall activity level reduced to 70% of expected. Unable to perform strenuous duties, but able to perform light duty or deskwork 4 - 5 hours a day, but requires rest periods. Has to rest/sleep 1-2 hours daily.

**40**: Moderate symptoms at rest. Moderate to severe symptoms with exercise or activity. Overall activity level reduced to 50-70% of expected. Able to go out once or twice a week. Unable to perform strenuous duties. Able to work sitting down at home 3-4 hours a day, but requires rest periods.

**30**: Moderate to severe symptoms at rest. Severe symptoms with any exercise. Overall activity level reduced to 50% of expected. Usually confined to house. Unable to perform any strenuous tasks. Able to perform deskwork 2-3 hours a day, but requires rest periods.

**20**: Moderate to severe symptoms at rest. Unable to perform strenuous activity. Overall activity 30-50% of expected. Unable to leave house except rarely. Confined to bed most of day. Unable to concentrate for more than 1 hour a day.

**10**: Severe symptoms at rest. Bed ridden the majority of the time. No travel outside of the house. Marked cognitive symptoms preventing concentration.

**0**: Severe symptoms on a continuous basis. Bed ridden constantly, unable to care for self.

# COMPASS 31 [5]

1. In the past year, have you ever felt faint, dizzy, “goofy”, or had difficulty thinking soon after standing up from a sitting or lying position?

   1 Yes
   2 No (if you marked No, please skip to question 5)

2. When standing up, how frequently do you get these feelings or symptoms?

   1 Rarely
   2 Occasionally
   3 Frequently
   4 Almost Always

3. How would you rate the severity of these feelings or symptoms?

   1 Mild
   2 Moderate
   3 Severe

4. In the past year, have these feelings or symptoms that you have experienced:

   1 Gotten much worse
   2 Gotten somewhat worse
   3 Stayed about the same
   4 Gotten somewhat better
   5 Gotten much better
   6 Completely gone

5. In the past year, have you ever noticed color changes in your skin, such as red, white, or purple?

   1 Yes
   2 No (if you marked No, please skip to question 8)

6. What parts of your body are affected by these color changes? (Check all that apply)

   1 Hands
   2 Feet

7. Are these changes in your skin color:

   1 Getting much worse
   2 Getting somewhat worse
   3 Staying about the same
   4 Getting somewhat better
   5 Getting much better
   6 Completely gone

8. In the past 5 years, what changes, if any, have occurred in your general body sweating?

   1 I sweat much more than I used to
   2 I sweat somewhat more than I used to
   3 I haven’t noticed any changes in my sweating
   4 I sweat somewhat less than I used to
   5 I sweat much less than I used to

9. Do your eyes feel excessively dry?

   1 Yes
   2 No

10. Does you mouth feel excessively dry?

   1 Yes
   2 No

11. For the symptom of dry eyes or dry mouth that you have had for the longest period of time, is this symptom:

1 I have not had any of these symptoms
2 Getting much worse
3 Getting somewhat worse
4 Staying about the same
5 Getting somewhat better
6 Getting much better
7 Completely gone

12. In the past year, have you noticed any changes in how quickly you get full when eating a meal?

1 I get full a lot more quickly now than I used to
2 I get full more quickly now than I used to
3 I haven't noticed any change
4 I get full less quickly now than I used to
5 I get full a lot less quickly now than I used to

13. In the past year, have you felt excessively full or persistently full (bloated feeling) after a meal?

1 Never
2 Sometimes
3 A lot of the time

14. In the past year, have you vomited after a meal?

1 Never
2 Sometimes
3 A lot of the time

15. In the past year, have you had a cramping or colicky abdominal pain?

1 Never
2 Sometimes
3 A lot of the time

16. In the past year, have you had any bouts of diarrhea?

1 Yes
2 No (if you marked No, please skip to question 20)

17. How frequently does this occur?

1 Rarely
2 Occasionally
3 Frequently ____________ times per month
4 Constantly

18. How severe are these bouts of diarrhea?

1 Mild
2 Moderate
3 Severe

19. Are your bouts of diarrhea getting:

1 Much worse
2 Somewhat worse
3 Staying the same
4 Somewhat better
5 Much better
6 Completely gone

20. In the past year, have you been constipated?

1 Yes
2 No (if you marked No, please skip to question 24)

21. How frequently are you constipated?

1 Rarely
2 Occasionally
3 Frequently ____________ times per month
4 Constantly

22. How severe are these episodes of constipation?

1 Mild
2 Moderate
3 Severe

23. Is your constipation getting:

1 Much worse
2 Somewhat worse
3 Staying the same
4 Somewhat better
5 Much better
6 Completely gone

24. In the past year, have you ever lost control of your bladder function?

1 Never
2 Occasionally
3 Frequently ____________ times per month
4 Constantly

25. In the past year, have you had difficulty passing urine?

1 Never
2 Occasionally
3 Frequently ____________ times per month
4 Constantly

26. In the past year, have you had trouble completely emptying your bladder?

1 Never
2 Occasionally
3 Frequently ____________ times per month
4 Constantly

27. In the past year, without sunglasses or tinted glasses, has bright light bothered your eyes?

1 Never (if you marked Never, please skip to question 29)
2 Occasionally
3 Frequently
4 Constantly

28. How severe is this sensitivity to bright light?

1 Mild
2 Moderate
3 Severe

29. In the past year, have you had trouble focusing your eyes?

1 Never (if you marked Never, please skip to question 31)
2 Occasionally
3 Frequently
4 Constantly

30. How severe is this focusing problem?

1 Mild
2 Moderate
3 Severe

31. Is the most troublesome symptom with your eyes (i.e. sensitivity to bright light or trouble focusing) getting:

1 I have not had any of these symptoms
2 Much worse
3 Somewhat worse
4 Staying about the same
5 Somewhat better
6 Much better
7 Completely gone

COMPASS31-Scoring Algorithm. The raw domain scores are derived by adding the points for the questions comprising each domain. Where an answer to a question is not assigned a point, the score for that answer is zero. The final domain scores are generated by multiplying the raw score with a weight index (see Table 3). The total score is the sum of all domain scores.

| **Domain** | **Item** | **Answer** | **Points** |
|---|---|---|---|
| Orthostatic Intolerance | | | |
| | 1 | 1 | 1 |
| | 2 | 2 | 1 |
| | | 3 | 2 |
| | | 4 | 3 |
| | 3 | 1 | 1 |
| | | 2 | 2 |
| | | 3 | 3 |
| | 4 | 1 | 3 |
| | | 2 | 2 |
| | | 3 | 1 |
| Vasomotor | | | |
| | 5 | 1 | 1 |
| | 6 | 1 | 1 |
| | | 2 | 1 |
| | 7 | 1 | 3 |
| | | 2 | 2 |
| | | 3 | 1 |
| Secretomotor | | | |
| | 8 | 1 | 1 |
| | | 4 | 1 |
| | | 5 | 2 |
| | 9 | 1 | 1 |
| | 10 | 1 | 1 |
| | 11 | 2 | 3 |
| | | 3 | 2 |
| | | 4 | 1 |
| Gastrointestinal | | | |
| | 12 | 1 | 2 |
| | | 2 | 1 |
| | 13 | 2 | 1 |
| | | 3 | 2 |
| | 14 | 2 | 1 |
| | | 3 | 2 |
| | 15 | 2 | 1 |
| | | 3 | 2 |
| | 16 | 1 | 1 |
| | 17 | 2 | 1 |
| | | 3 | 2 |
| | | 4 | 3 |
| | 18 | 1 | 1 |

| | | | |
|---|---|---|---|
| | | 2 | 2 |
| | | 3 | 3 |
| | 19 | 1 | 3 |
| | | 2 | 2 |
| | | 3 | 1 |
| | 20 | 1 | 1 |
| | 21 | 2 | 1 |
| | | 3 | 2 |
| | | 4 | 3 |
| | 22 | 1 | 1 |
| | | 2 | 2 |
| | | 3 | 3 |
| | 23 | 1 | 3 |
| | | 2 | 2 |
| | | 3 | 1 |
| Bladder | | | |
| | 24 | 2 | 1 |
| | | 3 | 2 |
| | | 4 | 3 |
| | 25 | 2 | 1 |
| | | 3 | 2 |
| | | 4 | 3 |
| | 26 | 2 | 1 |
| | | 3 | 2 |
| | | 4 | 3 |
| Pupillomotor | | | |
| | 27 | 2 | 1 |
| | | 3 | 2 |
| | | 4 | 3 |
| | 28 | 1 | 1 |
| | | 2 | 2 |
| | | 3 | 3 |
| | 29 | 2 | 1 |
| | | 3 | 2 |
| | | 4 | 3 |
| | 30 | 1 | 1 |
| | | 2 | 2 |
| | | 3 | 3 |
| | 31 | 2 | 3 |
| | | 3 | 2 |
| | | 4 | 1 |

# Assessment of POTS

**Name:** __________________

**Date:** __________________

**Pulse and blood pressure in sitting position and standing**

| | Time | RR | Pulse |
|---|---|---|---|
| Sitting positin | | | |
| Standing 0’ | | | |
| Standing 2’ | | | |
| Standing 5’ | | | |
| Standingd 10’ | | | |
| Feeling after 10 minutes | | | |

POTS is defined as an increase of heart rate pulse from the resting position to a standing position of more than 30 beats per minute or a pulse of more than 120 beats per minute accompanied by symptoms such as fainting, blurry vision, nausea etc.
Orthostatic hypotension is defined as a drop of 20mmHg in systolic or a drop of 10mmHg in diastolic blood pressure in the first three minutes of standing upright.

# Hand Strength Test

Patient ID

Date:

- ○ male
- ○ female

- ○ right-handed
- ○ left-handed

| Timepoint | 1 | 2 | 3 | 4 | 5 | 6 | 7 | 8 | 9 | 10 |
|---|---|---|---|---|---|---|---|---|---|---|
| 0 (1) | | | | | | | | | | |
| 60‘ (2) | | | | | | | | | | |

Fmax1 maximal value of 10 pulls at timepoint 0
Fmax2 maximal value of 10 pulls at timepoint 60 minutes

Fmean1 mean value of 10 pulls at timepoint 0
Fmean2 mean value of 10 pulls at timepoint 60 minutes

## Supplementary References


1. Carruthers, B.M., et al. Myalgic Encephalomyelitis/Chronic Fatigue Syndrome. Journal of Chronic Fatigue Syndrome 11, 7-115 (2003).
2. Cotler, J., Holtzman, C., Dudun, C. & Jason, L.A. A Brief Questionnaire to Assess Post-Exertional Malaise. *Diagnostics (Basel)* **8**, 66 (2018).
3. Cella, M. & Chalder, T. Measuring fatigue in clinical and community settings. *Journal of psychosomatic research* **69**, 17-22 (2010).
4. Bell, D.S. The doctor’s guide to chronic fatigue syndrome. (Addison-Wesley Publishing Co, 1995).
5. Sletten, D.M., Suarez, G.A., Low, P.A., Mandrekar, J. & Singer, W. COMPASS 31: a refined and abbreviated Composite Autonomic Symptom Score. *Mayo Clin Proc* **87**, 1196-1201 (2012).
