# Supplementary material for: A prospective observational study of post-COVID-19 chronic fatigue syndrome following the first pandemic wave in Germany and biomarkers associated with symptom severity
Source: Nat Commun. 2022 Aug 30;13:5104. doi: 10.1038/s41467-022-32507-6 (PMC9426365; doi:10.1038/s41467-022-32507-6)
Supplement: Supplementary file 3 — Reporting Summary [file 41467_2022_32507_MOESM3_ESM.pdf]

nature portfolio

Corresponding author(s): NCOMMS-21-21045E

Last updated by author(s): Jun 27, 2022

Reporting Summary

Nature Portfolio wishes to improve the reproducibility of the work that we publish. This form provides structure for consistency and transparency in reporting. For further information on Nature Portfolio policies, see our Editorial Policies and the Editorial Policy Checklist.

Statistics

For all statistical analyses, confirm that the following items are present in the figure legend, table legend, main text, or Methods section.

n/a	Confirmed	
☐	☒	The exact sample size (*n*) for each experimental group/condition, given as a discrete number and unit of measurement
☐	☒	A statement on whether measurements were taken from distinct samples or whether the same sample was measured repeatedly
☐	☒	The statistical test(s) used AND whether they are one- or two-sided<br>*Only common tests should be described solely by name; describe more complex techniques in the Methods section.*
☐	☒	A description of all covariates tested
☐	☒	A description of any assumptions or corrections, such as tests of normality and adjustment for multiple comparisons
☐	☒	A full description of the statistical parameters including central tendency (e.g. means) or other basic estimates (e.g. regression coefficient) AND variation (e.g. standard deviation) or associated estimates of uncertainty (e.g. confidence intervals)
☐	☒	For null hypothesis testing, the test statistic (e.g. *F*, *t*, *r*) with confidence intervals, effect sizes, degrees of freedom and *P* value noted<br>*Give P values as exact values whenever suitable.*
☒	☐	For Bayesian analysis, information on the choice of priors and Markov chain Monte Carlo settings
☒	☐	For hierarchical and complex designs, identification of the appropriate level for tests and full reporting of outcomes
☐	☒	Estimates of effect sizes (e.g. Cohen's *d*, Pearson's *r*), indicating how they were calculated

Our web collection on statistics for biologists contains articles on many of the points above.

Software and code

Policy information about availability of computer code

Data collection	All data was recorded using the Research Electronic Data Capture (REDCap) database
Data analysis	n/a

For manuscripts utilizing custom algorithms or software that are central to the research but not yet described in published literature, software must be made available to editors and reviewers. We strongly encourage code deposition in a community repository (e.g. GitHub). See the Nature Portfolio guidelines for submitting code & software for further information.

Data

Policy information about availability of data

All manuscripts must include a data availability statement. This statement should provide the following information, where applicable:

- Accession codes, unique identifiers, or web links for publicly available datasets
- A description of any restrictions on data availability
- For clinical datasets or third party data, please ensure that the statement adheres to our policy

n/a

Human research participants

Policy information about studies involving human research participants and Sex and Gender in Research.

Reporting on sex and gender	n/a
Population characteristics	see below
Recruitment	see below
Ethics oversight	This study is part of the Pa-COVID-19 study of the Charité 19 and approved by the Ethics Committee of the Charité - Universitätsmedizin Berlin in accordance with the 1964 Declaration of Helsinki and its later amendments (EA2/066/20)

Note that full information on the approval of the study protocol must also be provided in the manuscript.

Field-specific reporting

Please select the one below that is the best fit for your research. If you are not sure, read the appropriate sections before making your selection.

☒ Life sciences ☐ Behavioural & social sciences ☐ Ecological, evolutionary & environmental sciences

For a reference copy of the document with all sections, see nature.com/documents/nr-reporting-summary-flat.pdf

Life sciences study design

All studies must disclose on these points even when the disclosure is negative.

Sample size	42 patients were included in this observational study. Patients were selected based on a screening questionnaire with mild to moderate COVID-19 and persistent symptoms of moderate to severe fatigue and exertion intolerance six months post-infection in the absence of relevant cardiac, respiratory, neurological, or psychiatric comorbidty.
Data exclusions	Patients with relevant cardiac, respiratory, neurological, or psychiatric comorbidty and in the absence of PCR or serology (SARS-CoV-2 IgG/IgA), or typical initial symptoms including loss of smell and taste.
Replication	Source data are provided with this paper. Pseudonymized patient data may be available upon written reasonable request to the corresponding author (CK). Additional documents including the protocol synopsis and informed consent form are available as supplementary files within the original submission.
Randomization	n/a
Blinding	n/a

Reporting for specific materials, systems and methods

We require information from authors about some types of materials, experimental systems and methods used in many studies. Here, indicate whether each material, system or method listed is relevant to your study. If you are not sure if a list item applies to your research, read the appropriate section before selecting a response.

Materials & experimental systems

n/a	Involved in the study	
☒	☐	Antibodies
☒	☐	Eukaryotic cell lines
☒	☐	Palaeontology and archaeology
☒	☐	Animals and other organisms
☐	☒	Clinical data
☒	☐	Dual use research of concern

Methods

n/a	Involved in the study	
☒	☐	ChIP-seq
☒	☐	Flow cytometry
☒	☐	MRI-based neuroimaging

Clinical data

Policy information about clinical studies

All manuscripts should comply with the ICMJE guidelines for publication of clinical research and a completed CONSORT checklist must be included with all submissions.

Clinical trial registration	Pa-COVID-19 is registrated at the German Clinical Trials Register and WHO International Clinical Trials Registry Platform (DRKS00021688)
Study protocol	https://pubmed.ncbi.nlm.gov/32535877/
Data collection	Data collection began in August seventh 2020 and for this manuscript final date was November 27th 2020, the study is ongoing
Outcomes	n/a.
